# Active video games as an adjunct to pulmonary rehabilitation of patients with Chronic Obstructive Pulmonary Disease: a commentary on a systematic review

**DOI:** 10.1097/PHM.0000000000001341

**Published:** 2024

**Authors:** Catherine Harris, Jenna Banks, Catherine Edwards, Sarah Hurst, James Edward Hill

**Affiliations:** 1 Health Technology Assessment Unit, University of Central Lancashire, Preston; 2 Lancashire Teaching Hospitals NHS Foundation Trust, Preston; 3 Lancashire and South Cumbria NHS Foundation Trust, Lancashire

**Keywords:** Pulmonary Rehabilitation, Active Video Games, Chronic Obstructive Pulmonary Disease, systematic reviews

## Abstract

Pulmonary rehabilitation is a key evidence-based intervention to improve the outcomes of people living with Chronic Obstructive Pulmonary Disease (COPD). However, there are challenges in delivering pulmonary rehabilitation including reduced referral rates and suboptimal uptake and completion rates. Active video game interventions, when used as an adjunct, may increase the adoption of and access to pulmonary rehabilitation. This commentary summarises and critically appraises a systematic review which investigated the effectiveness of active video games as a supplementary component in the pulmonary rehabilitation of individuals suffering from chronic obstructive pulmonary disease.

## Introduction

Chronic Obstructive Pulmonary Disease (COPD) is a common progressive lung condition causing persistent breathing difficulties in patients [1]. Many patients with COPD experience a significant burden of symptoms and exacerbations in their condition which impacts on their quality of life and results in increased use of healthcare resources [2].

Pulmonary rehabilitation is a key evidence-based intervention to improve the outcomes of people living with COPD, improve their quality of life and reduce healthcare utilisation [3]. Current challenges in delivering pulmonary rehabilitation include reduced referral rates [4], and suboptimal uptake and completion rates [5].

The NHS Long Term Plan supports providers of pulmonary rehabilitation to develop new models of care to increase accessibility and to promote self-management and personalised care [6]. The expansion of digital healthcare options through the COVID-19 pandemic also encourages the exploration of digital solutions to complement the traditional face-to-face model of pulmonary rehabilitation [7]. One such digital solution which may have the potential to enhance access to, and boost the adoption of, rehabilitation is the use of active video games [8]. In a prior systematic review conducted by Wang et al. in 2020 [8], they investigated the effectiveness of active video games as a supplementary component in the pulmonary rehabilitation of individuals suffering from COPD.

### Aim of commentary

This commentary aims to critically appraise the methods used within the review by Wang et al., 2020 [8], and expand upon the findings in the context of clinical practice.

## Methods of Wang et al., (2020)

A comprehensive search of seven databases was carried out to 3 April 2019. Randomised controlled trials (RCTs) and quasi-experimental studies of active video gaming as an intervention for the pulmonary rehabilitation of patients with an objective diagnosis of COPD were included in the review. Robust screening, data extraction and quality assessment processes were undertaken by two reviewers independently. Quality assessment was conducted using the Cochrane Risk of Bias tool for RCTs, and the Methodological Index for Nonrandomized Studies (MINORS) for quasi-experimental studies. A meta-analysis was conducted using a random effects model for the outcome of exercise capacity (measured by the 6-min walk distance test). The outcomes of dyspnoea, quality of life, enjoyment, adherence, and adverse events were synthesised through a descriptive analysis.

## Results of Wang et al., (2020)

Seven papers were included in the review: three RCTs and four quasi-experimental studies. For the three RCTs there were risk of bias concerns around blinding of participants and personnel, blinding of outcome assessment, and allocation concealment. For the four quasi-experimental studies there were risk of bias concerns around insufficient information on the outcome assessments in all four studies, lack of sample size calculations in three of the studies, and lack of an appropriate follow-up period in two of the studies. The review found that active video games as an adjunct to pulmonary rehabilitation, compared to pulmonary rehabilitation alone, produced a clinically and statistically significant increase in exercise capacity, with an average increase of 30.9 metres in the 6MWD test (95% Confidence Interval 10.63 to 51.16, P = 0.003) based on the results of the three RCTs. No heterogeneity was found in the outcome of the 6MWD test. However, the I^2^ test was not statistically significant.

In the descriptive analysis, none of the four studies reporting on level of dyspnoea found a statistically significant difference in the dyspnoea score in the active video game intervention groups. A significant improvement in quality of life after active video game interventions was found in four studies, and four studies reporting on enjoyment identified indications that patients found the active videogames to be enjoyable.

Two studies reported on adherence with the active videogame interventions. In one study adherence was self-recorded so the accuracy of the data could not be verified; another study determined adherence based on an attendance rate above 50% (76% in the study). Two studies reported on adverse events, with one reporting no adverse events occurring during the study, and the other reporting adverse events in six patients (the need to use nitroglycerin spray in one patient and temporary decrease in SpO_2_ below 85% in five patients) but did not report whether these occurred in the intervention or in the control groups.

## Commentary

Using the AMSTAR2 tool [9] to assess the quality of the review, 11 out of the 16 criteria were judged to be satisfactory. The criteria that were judged not to be satisfactory included no explicit statement that there was a review methods protocol prior to conducting the review, which introduces the possibility of reporting bias since it is not possible to compare the results of the review with what was originally intended [10]. Secondly, although the review authors did assess the risk of bias in the included studies, they did not interpret the potential impact of this on the certainty of the findings. Additionally, they did not investigate potential publication bias which is important to consider since missing studies can potentially skew the estimate of effects [11]. Finally, the sources of funding for each study were not reported. Due to the critical nature of these quality domains the results of the review should be interpreted with caution.

The main outcome analysed in the review was exercise capacity, as measured by the 6-minute walk distance test (6MWT). For patients with COPD the minimum difference in the 6MWT considered to be clinically important is 54m [12]. The results of the meta-analysis estimated an increase in the 6MWT much lower than this with an average of 30.9m, and with a difference of only 10.63m at the lowest point of the estimate. Therefore, although the evidence shows a positive effect, it is not significant enough to recommend a change to clinical practice. Additionally, the review found no evidence of statistically significant changes in the symptom of dyspnoea when comparing interventions incorporating active video games to conventional therapies alone, and therefore no conclusions can be drawn as to which is better to improve this symptom. This is reflected in other recent systematic reviews of video gaming interventions for other related conditions, such as general respiratory conditions [13] and cardiovascular disease [14], which have similarly found no clear difference in effect on exercise capacity or dyspnoea outcomes when comparing video game-based interventions with traditional rehabilitation.

However, studies included in this review indicated that participants found the active video game interventions to be enjoyable; an aspect which may encourage patient adherence to rehabilitation and engage patients to be more physically active. Guidelines recommend that patients with COPD should be encouraged to exercise as part of pulmonary rehabilitation [12, 15] and enjoyment has been identified as a key factor which may increase adherence to physical exercise in patients with chronic diseases [16]. Studies included in the review did not report any significant adverse events in participants using active video games suggesting that they are relatively safe to use (although clinical and patient safety was not the main aim of the included studies and future research should assess this further). Therefore, clinicians could consider suggesting active video games as a supplementary component to traditional pulmonary rehabilitation to encourage physical activity in patients, where they are readily accessible, and patients have an interest in using them. Active video games should only be used as an adjunct and not as a replacement for traditional pulmonary rehabilitation.

Another key aim of pulmonary rehabilitation is to maximise patients’ self-management of their condition and long-term adherence to positive health behaviours [15]. Although the review did report on adherence to the interventions it found that this was not robustly measured or reported by the included studies. Therefore, future research should further investigate patient levels of activation and compliance with rehabilitative strategies that incorporate active video games and should evaluate these outcomes over an extended follow-up period to assess maintenance over the longer term.

Four of the studies included in the review reported exercise intensity (as assessed by the Borg Dyspnea scale, with the scores ranging from 3 to 6); but three did not. Exercise intensity delivered by active video games is likely to vary depending on the system used, type of game, and on individual patient factors [17]. This may make it more challenging for clinicians to manage the intensity of exercise required at different levels when using active video games as an adjunct. Therefore, more detailed reporting of exercise intensity in research studies would be beneficial to support optimal and safe exercise prescription of these interventions.

The findings of this review are limited by the small number of studies being included (seven) with small sample sizes (10-60 participants). It would therefore be beneficial to conduct larger randomised controlled trials of active video game interventions for pulmonary rehabilitation to strengthen the evidence in this area. It is also notable that the severity of participant COPD in the included studies was moderate to very severe. Further research on the effectiveness of active video games in the mild COPD population would be valuable as active video game interventions may have the potential to increase the accessibility to rehabilitation for this group.

## Key Points

- Evidence from this review suggests that active video game interventions, when used as an adjunct to rehabilitation, have a positive but small effect on increasing exercise capacity for people with moderate to severe COPD.
- Clinicians could consider suggesting active video games as a supplementary component to rehabilitation to encourage patients to be more physically active where they are readily accessible, and patients have an interest in using them.
- Active video games should only be used as an adjunct and not as a replacement for traditional pulmonary rehabilitation.
- When suggesting the use of active video games as an adjunct to rehabilitation, consideration should be given to individual patients’ levels of activation and digital literacy.
